# Utilizing the σ-complex stability for quantifying reactivity in nucleophilic substitution of aromatic fluorides

**DOI:** 10.3762/bjoc.9.90

**Published:** 2013-04-23

**Authors:** Magnus Liljenberg, Tore Brinck, Tobias Rein, Mats Svensson

**Affiliations:** 1 AstraZeneca, S-151 85 Södertälje, Sweden; 2 Applied Physical Chemistry, KTH Royal Institute of Technology, S-100 44 Stockholm, Sweden, Fax: +46 8 790 8207, Tel: +46 8 790 8210

**Keywords:** computational, DFT, nucleophilic aromatic substitution, reactivity, substrate selectivity, reactive intermediates

## Abstract

A computational approach using density functional theory to compute the energies of the possible σ-complex reaction intermediates, the “σ-complex approach”, has been shown to be very useful in predicting regioselectivity, in electrophilic as well as nucleophilic aromatic substitution. In this article we give a short overview of the background for these investigations and the general requirements for predictive reactivity models for the pharmaceutical industry. We also present new results regarding the reaction rates and regioselectivities in nucleophilic substitution of fluorinated aromatics. They were rationalized by investigating linear correlations between experimental rate constants (*k*) from the literature with a theoretical quantity, which we call the sigma stability (*SS*). The *SS* is the energy change associated with formation of the intermediate σ-complex by attachment of the nucleophile to the aromatic ring. The correlations, which include both neutral (NH_3_) and anionic (MeO^−^) nucleophiles are quite satisfactory (*r* = 0.93 to *r* = 0.99), and *SS* is thus useful for quantifying both global (substrate) and local (positional) reactivity in S_N_Ar reactions of fluorinated aromatic substrates. A mechanistic analysis shows that the geometric structure of the σ-complex resembles the rate-limiting transition state and that this provides a rationale for the observed correlations between the *SS* and the reaction rate.

## Introduction

### Background

Computational chemistry has become an indispensible tool for medicinal chemists, biologists and pharmacologists throughout all stages of the pharmaceutical research process. One application is in the selection or virtual screening of the, in many cases, numerous possible alternative synthetic routes to a target molecule, e.g., a candidate drug. To this end, efficient models that enable the prediction of product selectivity and relative reaction rates in a quantitative or semiquantitative way would be highly valuable. As a complement to experimental work, the use of such tools can help to minimize “trial and error” experimentation. However, one condition for such a tool to be of practical value is that there is a balance between accuracy and throughput: the ideal model should give a sufficiently high accuracy over as large a synthetic space as possible while at the same time allowing a high throughput and robustness. The main interest, and also the original reason for this work, was to investigate whether it is possible to develop a predictive reactivity model that could combine high throughput with a quantitative or semiquantitative accuracy.

### Predictive reactivity models

In pharmaceutical research there is a need to run large substance libraries through the virtual screening procedures, and this has set a number of boundary conditions or guiding principles for the predictive reactivity models:

**1. Generality**. The ideal method should be applicable to as large a reactant space as possible. It should at least not depend on previously determined experimental results for the specific, narrow subset of reactants presently under study.

**2. Accuracy**. The predictive models developed would have to have an accuracy that is high enough for the medicinal chemist to be able to judge a reaction as suitable or not for further investigations. From a regioselectivity perspective, for example, this means that it is not sufficient to simply be able to predict the probable main site for electrophilic or nucleophilic attack: it would also be very advantageous to be able to sort out any reaction that is likely to give a (hard to separate) mixture of isomers for the coming process.

**3. Throughput**. On the other hand, elaborate and time-consuming modeling of reactions that closely reproduce the experimental results would be too costly. It is of little value to know whether a regioisomeric mixture of two isomers for a reaction is likely to be a 70:30 or a 60:40 mixture; on the other hand, it is of key importance to predict whether a reaction will give a 70:30 or a 99:1 mixture.

**4. Robustness**. It would be a great advantage if the procedures suggested could be scripted and the computations could be performed automatically as “black box” calculations, with the input being large substance libraries. To this end it, would be advantageous if one could chose relevant structures on the potential energy surface (PES) that have large convergence radii for default starting structures, that is, where the geometry optimization is fairly insensitive to the exact starting geometry. It would also be an advantage if the procedure did not require a highly trained expert to perform the calculations, but was instead within the reach of, e.g., a synthetic chemist.

There are two main types of theoretical assessment of reactivity: property- or descriptor-based and direct modeling of the PES, especially of the rate-determining TS. The first type is generally termed QSAR (quantitative structure–activity relationships), where experimentally known or calculated properties are fitted to observed reactivities. These models frequently utilize descriptors derived from the reactants and they also require access to experimental data from substances of roughly the same type as those that are to be predicted [1]. Many of the property- or descriptor-based methods are fast and robust, but inherent is a poorer accuracy since they do not take the relevant TS or solvation into account.

Quantum chemistry methods exploring the PES on the other hand can, with sufficient modeling and appropriate level of theory, yield very accurate results and do not rely on predetermined experimental data. However, this procedure is slow, computationally costly and in many cases difficult to automate, and it also requires manual input from an expert in computational chemistry. In summary the property- or descriptor-based methods typically fulfill the requirements 3 and 4 above but fail 1 and 2, while the TS methods typically fulfill the requirements 1 and 2 but fail 3 and 4.

There are approaches reported where successful attempts have been made to strike a compromise between generality/accuracy and throughput/robustness. One example is the so-called Q2MM method, which is designed to apply molecular mechanics calculations to transition states in chemical reactions, especially for predictive catalysis. It is particularly suitable for stereoselectivity calculations where there is a need to virtually screen large ligand libraries [1]. Another example are the so-called QM/MM methods [2], where part of the structure is treated with QM methods and other parts with MM methods, for example, in enzymes where the active site can be modeled with QM and the remaining structure with MM methods.

### Predictive models for the S_N_Ar reaction

This paper is a continuation of our work on the predictive computational modeling of the synthetically and industrially important S_N_Ar and S_E_Ar reactions (nucleophilic and electrophilic aromatic substitution, respectively) [3–5]. The putative mechanism for the S_N_Ar reaction involves attack of a nucleophile and the formation of an intermediate σ-complex (also called the Meisenheimer complex) followed by elimination of the leaving group [6]. In the case of attack of anionic nucleophiles (such as MeO^−^) on fluorinated aromatics, the intermediate σ-complex is anionic and the leaving group is F^−^, whereas in the case of neutral nucleophiles (such as NH_3_) the intermediate σ-complex is zwitterionic and the leaving group is HF. The departure of H and F can proceed along different mechanisms [7–9].

Several methods for predicting local reactivity, or regioselectivity, in S_N_Ar reactions have been reported. Among the earlier ones is the I_π_-repulsion theory based on calculating the fractional charge with Hückel theory [10–11], and an approach based on the frontier molecular orbital method [12]. More recent attempts include calculation based on Fukui indices [13], local softness and hardness reactivity descriptors [14], dual descriptors for both electrophilicity and nucleophilicity [15], and calculation of the thermodynamic stability of the σ-complex [4–5,16–17]. A number of theoretical studies have also been carried out that concern global or substrate reactivity in S_N_Ar reactions. In one paper the reactivity of two systems was computed, in order to elucidate the energy barriers in different mechanistic steps for these systems [18]. In another, a simple numerical method based on a few reactivity parameters was used to predict the relative reactivities in the reaction between methoxide anion and a series of chlorofluorobenzene derivatives [19]. In two other articles, the authors studied the effect of different substituents on the reactivity of one model substance. The first dealt with the Newman–Kwart rearrangement of an aromatic thionocarbamate by intramolecular nucleophilic aromatic substitution [20], the second applied semiempirical methods to study the reaction between methoxide anion and a number of 4-substituted 1-chloro-2-nitrobenzenes [21].

There are also many studies dealing with the formulation of empirical rules concerning reactivity in the nucleophilic substitution of fluorinated aromatics [22–25]. The nature of the empirical rules that typically emerge can be exemplified by the following (concerning perfluoroaromatic systems): “trifluoromethyl, like ring nitrogen, activates both ortho- and para-positions to a similar degree” [22]. Such rule-based mnemonics can be useful as qualitative tools, but tend to be highly case-specific and not quantitatively accurate. Should there, in this specific example, be a deactivating substituent on the system beside trifluoromethyl, or should the substituent be fluoromethyl instead of trifluoromethyl, then another empirical rule would have to be developed.

To the best of our knowledge, a more general reactivity treatment is still lacking: a treatment that can be of use on at least a semiquantitative basis with respect to both global and local reactivity, that is, substrate as well as positional selectivity, for a wider range of substrates and nucleophiles. In this study we have included both different carbocyclic and heterocyclic substrates as well as neutral and anionic nucleophiles.

The σ-complex intermediate in S_N_Ar reactions has been considered to be the crucial intermediate governing both reactivity and regioselectivity [26]. Our method is based on calculation of the relative stabilities of the σ-complex intermediates using density functional theory, and is intended for kinetically controlled reactions. We assume that their properties, rather than those of the initial aromatic reactants, are crucial in determining reactivity. We have successfully applied the σ-complex approach to predict regioselectivity in reactions of carbocyclic as well as heterocyclic fluorinated substrates [4–5]. Anionic nucleophiles (anions of methanol, benzyl alcohol and hydrogen sulfide) were investigated, as well as neutral nucleophiles (amines). The accuracy was well within 1 kcal/mol and the predictions can be used in a quantitative way. It was necessary to include polarizable continuum model (PCM) solvent calculations, either as an a posteriori single point calculation or by optimizing the structures directly in solvent, to obtain the accuracy at this level. Muir and Baker [16–17] have investigated the case of nucleophilic attack of anionic nucleophiles to aromatic fluorides for a great many examples. They found that an approach using F^−^ as model nucleophile without solvent calculations was sufficient to give a good qualitative prediction of the main site of nucleophilic attack. The σ-complex approach failed when the leaving group was Cl^−^/HCl or Br^−^/HBr both for anionic and neutral nucleopiles, because of difficulties in finding relevant σ-complex structures. Instead an approach where we assumed a concerted substitution step and used such transition-state structures gave quantitatively useful results [5]. Recent results by Fernandez et al [26] show that σ-complex structures can be found if the leaving group is bound to the ring through an element in the second row of the periodic table (i.e., –F, –NH_2_, –OH), but not (unless the structure is highly stabilized, e.g., by several nitro groups) if the element is from the third or fourth row (i.e., –Cl, –Br, sulfur groups).

In this study we introduce a concept that we call the Sigma Stability, *SS*, to also encompass global reactivity, that is, investigations dealing with the relative reactivity between different systems. This is the energy required for the formation of the σ-complex, *SS* = *E*_σ-complex_ − (*E*_aromate_ + *E*_nucleophile_). The purpose of the present study is to evaluate the scope and reliability of this widened application and to find the minimum level of theory and basis set necessary to strike a reasonable balance between the requirements 1–4 above. The success of this approach is based on a number of assumptions, which we have described in our previous work on S_N_Ar reactions [4–5].

The approach has an attractive feature in that it represents a distinct simplification compared to finding TS structures (as it mean optimizations to local minima), while preserving an accuracy that in many cases is sufficient for an, at least semiquantitative, prediction of reactivity.

## Results

The performance of the *SS* concept was examined on the basis of correlations with experimentally determined reaction rate constants (*k*) of three series of reactions (series A, B and C). The first series (series A) deals with the amination of nine different fluorinated heterocyclic substrates run at 25 °C. The second series (series B) with the amination of seven different carbocyclic substrates run at 80 °C. Except for the temperature, reaction series A and B were run under identical conditions, i.e., reaction of the substrate and ammonia in dioxane/water (60:40 v/v). The third series (series C) deals with the methoxylation of a series of 10 different polychlorofluorobenzenes run under identical conditions, i.e., reaction of the substrate and sodium methoxide in methanol at 50 °C. The experimental reaction rates span more than six orders of magnitude, both for the aminations and for the methoxylations.

**Figure 1:**
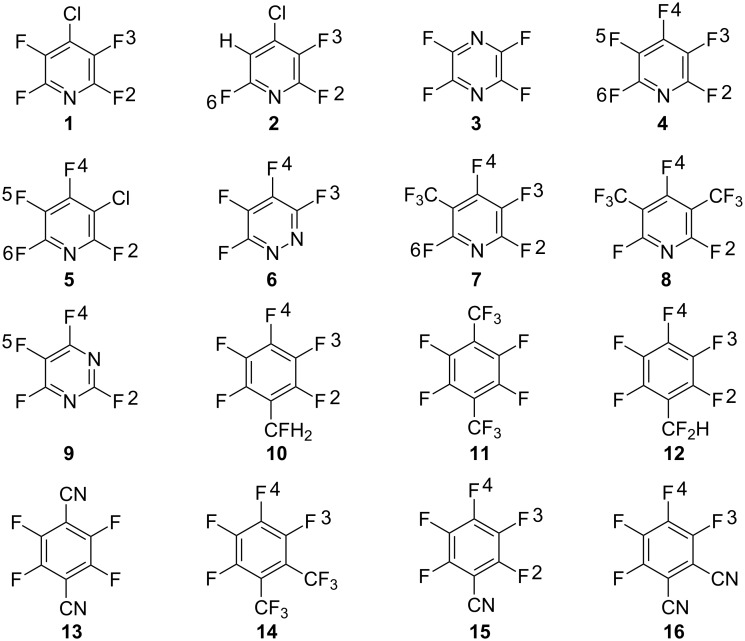
The structures investigated in the amination of heterocyclic and carbocyclic derivatives (series A and B, respectively) and the numbering of their positions.

**Figure 2:**
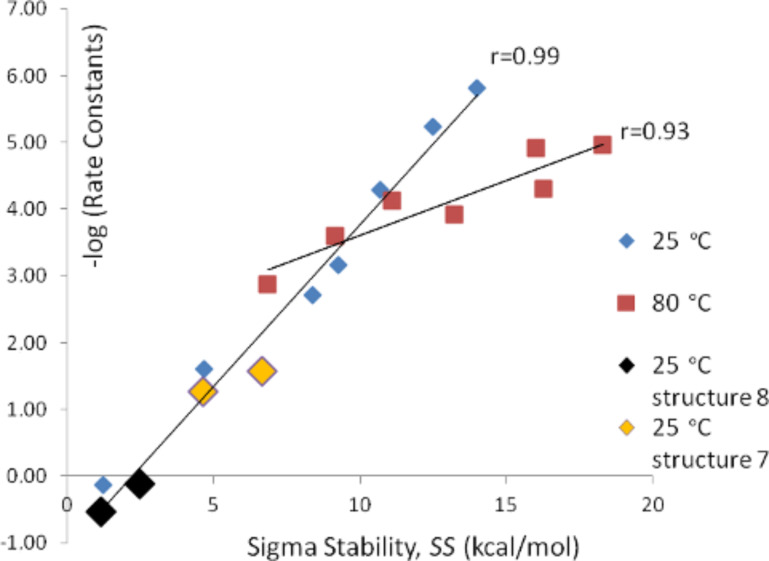
–log *k* as a function of *SS* in water for series A and B.

For the series of aminations (series A and B) the investigated structures and the numbering of positions are shown in Figure 1.The calculated *SS* values as well as experimental rate constants for series A and B are shown in Table 1 and Table 2, respectively. The correlation between *SS* values in water and experimental –log *k* values are shown in Figure 2. The *SS* values for the structures where experimental kinetic data are given for more than one positional isomer (Table 1, reactant **7**, entries 7 and 8; reactant **8**, entries 9 and 11) lie well on the correlation line in Figure 2 (these data points are indicated in the figure). The correlation coefficient is 0.99 for series A and 0.93 for series B. This corresponds to a mean average deviation of 0.5 kcal/mol and 1.4 kcal/mol, respectively. *SS* values based on in vacuo energies (obtained from the structures optimized in solvent) correlate poorly with the experimental –log *k* values [27]. In order to make a true kinetic prediction of an isomer in reaction series A that has not been experimentally observed in the literature, we calculated the *SS* value for position 3 in reactant **1**. This data is presented as entry 12 in Table 1 and it has, as might be expected, a very low predicted reaction rate constant. Experimental kinetic data were given at both 25 °C and 80 °C [28] for reactant **4** in Figure 1, and we tried to include this 80 °C data point in reaction series B, even though reactant **4** is a heterocyclic substrate (entry 8 in Table 2), but this made the correlation much poorer.

**Table 1:** Series A. Sigma stability (*SS*), experimental rate constants [28–29] and negative logarithms for experimental rate constants (−log *k*) for the aminations (with NH_3_) of different reactants in dioxane/water (60:40 v/v) at 25 °C. The structures of the reactants are shown in Figure 1.

Entry	Reactant (Figure 1)	Position of amination	*SS* (kcal mol^−1^) water	Reaction rate, *k* (l mol^−1^ s^−1^)^a^	−log *k*

1	**1**	2	14.00	1.55 × 10^−6^	5.81
2	**2**	2	12.47	5.92 × 10^−6^	5.23
3	**3**	Equal	10.70	5.07 × 10^−5^	4.29
4	**4**	4	9.26	6.80 × 10^−4^	3.17
5	**5**	4	8.37	1.92 × 10^−3^	2.72
6	**6**	4	4.67	2.52 × 10^−2^	1.60
7	**7**	2	6.66	2.66 × 10^−2^	1.58
8	**7**	4	4.63	5.31 × 10^−2^	1.27
9	**8**	2	2.46	1.31	−0.12
10	**9**	4	1.23	1.35	−0.13
11	**8**	4	1.15	3.39	−0.53
12	**1**	3	20.85	9.1 × 10^−10 b^	9.04^b^

^a^All rate constants are corrected for statistical factors (=degenerate positions). ^b^Value predicted from the regression line in Figure 2.

**Table 2:** Series B. Sigma stability (*SS*), experimental rate constants [22,28] and negative logarithms for experimental rate constants (−log *k*) for the amination (with NH_3_) of different reactants in dioxane/water (60:40 v/v) at 80 °C. The structures of the reactants are shown in Figure 1.

Entry	Reactant (Figure 1)	Position of amination	*SS* (kcal mol^−1^) water	Reaction rate, *k* (l mol^−1^ s^−1^)^a^	−log *k*

1	**10**	4	18.30	1.1 × 10^−5^	4.96
2	**11**	equal	16.02	1.23 × 10^−5^	4.91
3	**12**	4	16.26	5.0 × 10^−5^	4.30
4	**13**	equal	11.09	7.54 × 10^−5^	4.12
5	**14**	4	13.21	1.19 × 10^−4^	3.92
6	**15**	4	9.16	2.5 × 10^−4^	3.60
7	**16**	4	6.85	1.35 × 10^−3^	2.87
8^b^	**4**	4	9.26	2.8 × 10^−2^	1.55

^a^All rate constants are corrected for statistical factors (=degenerate positions). ^b^This entry is not part of the correlation of reaction series B, as it is a heterocyclic substrate.

**Figure 3:**
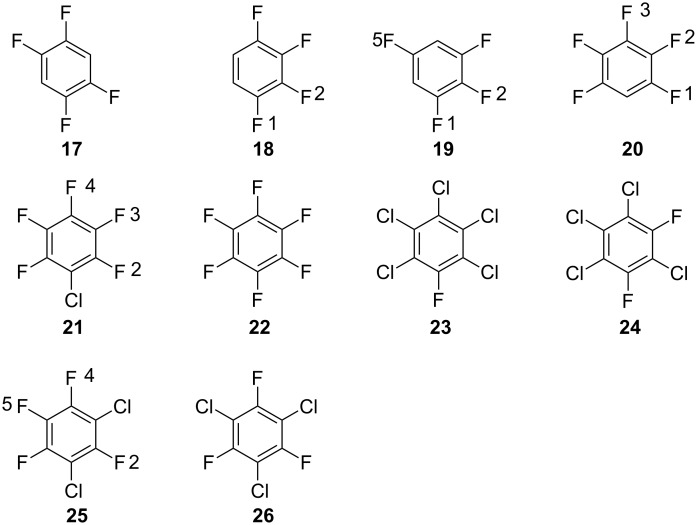
The structures investigated in the methoxylation of polychlorofluorobenzene derivatives (series C) and the numbering of their positions.

**Figure 4:**
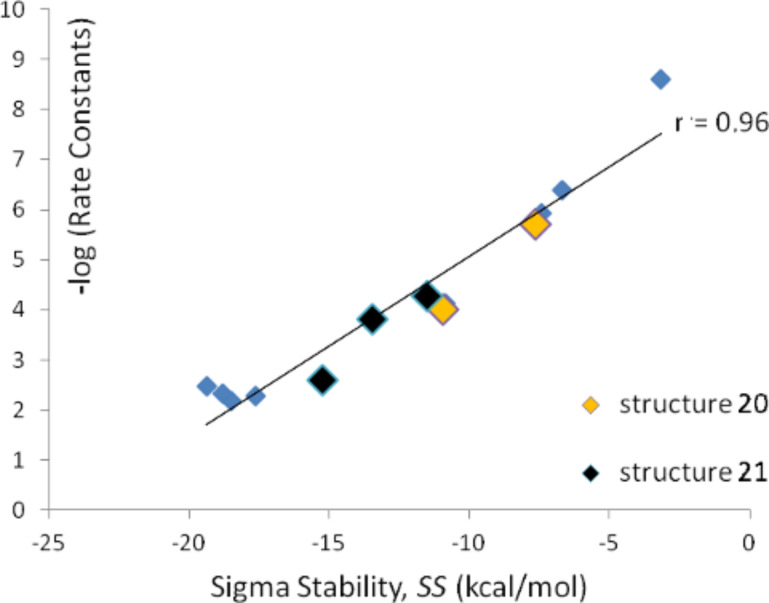
−log *k* as a function of *SS* in methanol for series C.

For the series of methoxylations (series C) the investigated structures and the numbering of positions are shown in Figure 3. The calculated *SS* values as well as experimental rate constants are shown in Table 3. The correlation between *SS* values in methanol and experimental –log *k* values are shown in Figure 4. The overall picture for this data set is quite analogous to the previous two and the *SS* values for the structures where experimental kinetic data are given for more than one positional isomer (Table 3, reactant **20**, entries 4 and 7; reactant **21**, entries 5, 8 and 9) falls well onto the correlation line in Figure 4 (these data points are indicated in the figure). The correlation coefficient is 0.96 for this data series, which corresponds to a mean average deviation of 1.1 kcal/mol. As for series A and B the *SS* values based on in vacuo energies show poor correlation coefficients with the experimental −log *k* values [27].

**Table 3:** Series C. Sigma stability (*SS*), experimental rate constants [19] and negative logarithms for experimental rate constants (−log *k*) for the reaction between sodium methoxide with different chlorofluorobenzene reactants in methanol at 50 °C. The structures of the reactants are shown in Figure 3.

Entry	Reactant (Figure 3)	Position of methoxylation	*SS* (kcal mol^−1^) methanol	Reaction rate, *k* (l mol^−1^ s^−1^)^a^	−log *k*

1	**17**	equal	−3.17	2.5 × 10^−9^	8.60
2	**18**	2	−6.71	4.0 × 10^−7^	6.40
3	**19**	1	−7.41	1.2 × 10^−6^	5.92
4	**20**	1	−7.63	2 × 10^−6 b^	5.70
5	**21**	3	−11.49	5.3 × 10^−5^	4.28
6	**22**	equal	−10.85	7.56 × 10^−5^	4.12
7	**20**	3	−10.96	1.01 × 10^−4^	4.00
8	**21**	2	−13.46	1.5 × 10^−4^	3.82
9	**21**	4	−15.24	2.52 × 10^−3^	2.60
10	**23**	One position	−19.39	3.3 × 10^−3^	2.48
11	**24**	equal	−18.82	4.8 × 10^−3^	2.32
12	**25**	4	−17.62	5.3 × 10^−3^	2.28
13	**26**	equal	−18.49	6.8 × 10^−3^	2.17

^a^All rate constants are corrected for statistical factors (=degenerate positions). ^b^Approximate value.

It would seem that the *SS* values for the methoxylation reactions are significantly lower than the true absolute energy values for forming the σ-complex and also that they are underestimated in a systematic fashion. The probable reason is that the solvation model used [30] systematically overestimates the solvation energy for the anionic σ-complexes. Experimental solvation energy values for σ-complexes are scarce, but Dillow and Kebarle [31] give the value −58 kcal/mol for the σ-complex resulting from the reaction between F^−^ and C_6_F_6_ in DMSO. The solvation energy obtained from our calculations of entry 6 in Table 3 (which is also from C_6_F_6_ but with MeO^−^ instead of F^−^ as nucleophile and with MeOH instead of DMSO as solvent) was −73 kcal/mol, and all of the σ-complexes in Table 3 that do not contain chlorine substituents (entries 1–4 and 6–7 in Table 3) have a calculated solvation energy in the range −73 to −78 kcal/mol. The problem with continuum models that gravely overestimate the solvation energy for anionic species is well known [32]: one reason is probably the scarcity of relevant solvation-energy data for organic ions in organic solvents, used in the parametrization work. This systematic underestimation is, however, without practical importance if the purpose is only semiquantitative ranking of substrate and positional reactivity.

## Discussion

Our results show that the substrate and positional reactivity can be predicted from the computed energy of the σ-complex. Performing single-point calculations at the optimized geometries using larger basis sets, by adding diffuse functions, does not in an appreciable way improve the correlation. Thermodynamic corrections taken from frequency calculations (harmonic approximations) of key intermediates also have minor effects on the correlation. The comparatively moderate levels of theory and basis set used by us in this study yielded an accuracy of the *SS* values of around 1 kcal/mol, measured as mean average deviation. Thus, it seems that the level of theory employed, B3LYP/6-31G(d,p), is sufficient for the present purpose, which is to make semiquantitative rankings of substrate and positional reactivity between species that are run under the same reaction conditions. It is important to note that the *SS* values do not give an explicit indication of absolute energy barriers, and the approach is thus limited to reactivity comparisons where species are run under the same reaction conditions. It is also worth noting that the correlation became poor in the one case where we tried to mix carbocyclic and heterocyclic substrates in one reaction series (Table 2). Any reactivity comparisons between series must be done with the utmost caution.

**Figure 5:**
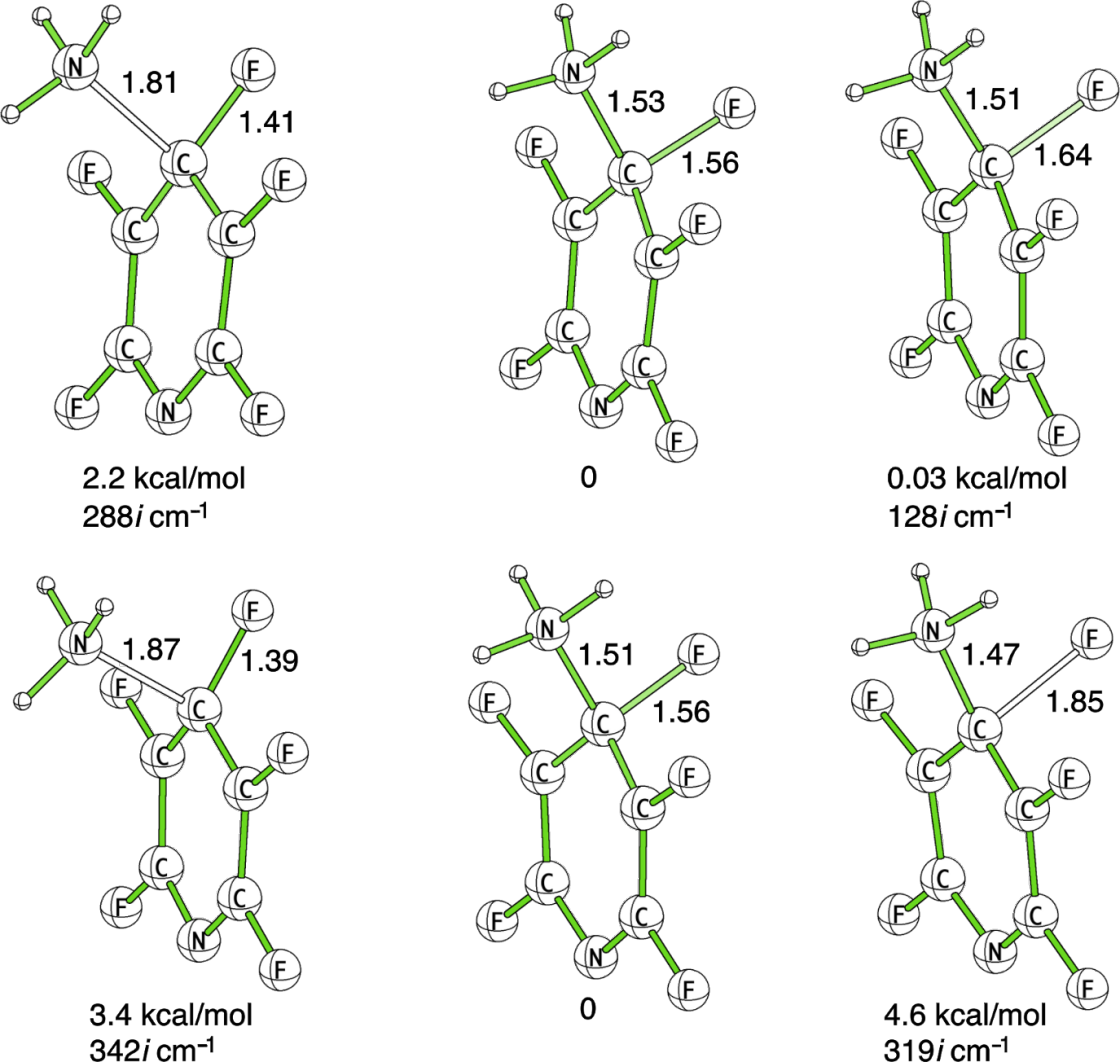
Optimized geometries, relative energies, and imaginary frequencies for the transition states and the σ-complex (middle structure) formed in the reaction between ammonia and **4**. The top and bottom rows show the results from optimizations at the B3LYP/6-31+G(d,p) and M06-2X/6-31+G(d,p) levels of theory, respectively.

In order to deduce the mechanistic basis behind the very good correlations between SS values and reaction rates, we computed the stationary points on the potential energy surface for the amination of **4.** We found that this characterization had to be performed using a larger basis set with diffuse functions to obtain accurate geometries and energy differences. The B3LYP/6-31+G(d,p) optimized geometries of TS1, the σ-complex and TS2 with PCM water solvation are depicted in Figure 5. All three structures are very similar, and the main difference between the σ-complex and TS2 is a slight elongation of less than 0.1 Å of the breaking C–F bond in TS2. An IRC-calculation showed that F leaves as F^−^ without the assistance of explicit hydrogen bonds from the NH_2_-group, and that the proton transfer to form HF takes place late in the concerted reaction step going from the σ-complex via TS2 to the products. An analysis of the energy differences between the three stationary points shows that the potential energy surface is extremely flat in the vicinity of the σ-complex. TS1 is only 2.2 kcal/mol higher in energy than the σ-complex, and TS2 has an almost identical energy to that of the σ-complex. The energy ordering of the two latter is even inversed after incorporation of zero-point vibrational energies. As we have already indicated, diffuse functions are important for obtaining an accurate potential energy surface for this reaction. This is not surprising, since during the process of going from TS1 via the σ-complex to TS2 the localization of negative charge at the fluorine atom is continuously increasing. Consequently, after removal of the diffuse functions the energy of TS1 relative to the σ-complex is decreased to 0.13 kcal/mol and the energy of TS2 is increased to 6.5 kcal/mol. Consistent with the picture that diffuse functions are needed to describe the negative charge formed at the fluorine, the C–F bond is much longer (1.83 Å) in the TS2 structure optimized without diffuse functions.

The fact that the σ-complex is very close both in energy and geometry to the rate-limiting transition state (TS1) provides a rationale for the very good correlations between *SS* values and reaction rates. However, a recent study has shown that the B3LYP functional predicts a concerted reaction in some cases, including an intramolecular S_N_Ar reaction, where the M06-2X functional and high level ab initio theory show that the reaction is stepwise [33]. In light of these observations, we investigated the potential energy surface also at the M06-2X/6-31+G(d,p) level of theory. The computed stationary points and their energies are depicted in Figure 5. The geometries are very similar to those obtained with the B3LYP functional. The largest difference is found for TS2, where the breaking C–F is extended from 1.64 to 1.85 Å with M06-2X. Observing the relative energies, we see much larger differences. TS2 is rate-determining with the M06-2X functional, and lies 4.6 kcal/mol higher in energy than the σ-complex. Also the relative energy of TS1 is raised, but only by 1.2 kcal/mol compared to B3LYP. Removing the diffuse functions from the basis set has a similar effect on the relative energies as in the B3LYP calculations; TS2 lies 12.8 kcal/mol above the σ-complex at the M06-2X/6-31G(d,p) level of theory.

On the basis of the current study, we cannot make any conclusive statement regarding whether TS1 or TS2 is rate-determing for S_N_Ar reactions with F as leaving group. In fact it may differ depending upon the nucleophile and the substrate. However, our analyses using both the B3LYP functional and the M06-2X functional strongly indicate that the structure of σ-complex is similar to the structure of the rate-limiting transition state, and that changes in the molecular structure that affect the relative energy of the transition state will affect the energy of the σ-complex to a similar extent. In light of these observations the good correlations between *SS* values and reaction rates are not surprising.

A distinct advantage with the σ-complex as model for the rate-limiting TS (apart from the more practical advantage that it is normally easier and faster to find computationally than the transition-state structure) is that there is no ambiguity in the structure of this stationary point on the potential-energy surface. Furthermore, our mechanistic analysis shows that a basis set with diffuse functions is necessary to describe the energy and structure of the rate-limiting transition state, and this increases the computational cost of a transition-state approach even further. It is tempting to investigate the generality of the *SS* concept further, by adding different nucleophiles, temperatures and reaction conditions, but this is at present unfortunately limited by the scarcity of experimental kinetic data.

## Conclusion

We have shown for several series of fluorinated aromatics that there exist good correlations between the relative σ-complex stabilization and experimental rate constants for S_N_Ar reactions involving both neutral and anionic nucleophiles. Thus, in combination with our previous studies, the present study demonstrates that the *SS* concept provides a methodology for the quantitative prediction of both regioselectivity and relative reactivity of S_N_Ar reactions of fluorinated substrates. Due to the low computational requirements and the potential for automatization, this method may find use in virtual reactivity screening in pharmaceutical research and development. We have further shown that the method has a solid mechanistic foundation, as the structure of the σ-complex is similar to the structure of the rate-determining transtion state of the reaction.

## Theoretical method

*SS* values were computed using DFT with the B3LYP hybrid functional and a 6-31G(d,p) basis set with effective core potentials on heavy atoms by using the Jaguar program [30]. Preliminary calculations showed that the zwitterionic σ-complex does not exist as a stationary point on the potential energy surface in vacuo. Therefore, all structures were optimized within the Poisson–Boltzmann finite-element solvation model (PBF) incorporated in the Jaguar software [30], using water as solvent for the amination reactions and methanol for the methoxylation reactions.

A more detailed analysis of the potential energy surface for the amination of one substrate was performed by geometry optimizations using the 6-31+G(d,p) basis set and the two functionals B3LYP and M06-2X. In these computations solvent effects were included by means of the polarizable continuum model as implemented in the Gaussian 09 suite of software [34]. All stationary points were characterized by means of frequency and IRC calculations.

## Supporting Information

File 1 Coordinates of all optimized structures, electronic energies, *SS* values calculated with larger basis set, and zero-point energies.
