# Supplementary material for: Utilizing the σ-complex stability for quantifying reactivity in nucleophilic substitution of aromatic fluorides
Source: Beilstein J Org Chem. 2013 Apr 23;9:791–9. doi: 10.3762/bjoc.9.90 (PMC3678587; doi:10.3762/bjoc.9.90)
Supplement: File 1 — Coordinates of all optimized structures, electronic energies, SS values calculated with larger basis set, and zero-point energies. [file Beilstein_J_Org_Chem-09-791-s001.pdf]

Supporting Information

for

Utilizing the σ-complex stability for quantifying reactivity in nucleophilic substitution of aromatic fluorides

Magnus Liljenberg,[*1] Tore Brinck,[*2] Tobias Rein[1], Mats Svensson[1]

Address: [1]AstraZeneca, S-151 85 Södertälje, Sweden and [2]Applied Physical Chemistry, KTH Royal Institute of Technology, S-100 44 Stockholm, Sweden, Fax: +46 8 790 8207, Tel: +46 8 790 8210

Email: Magnus Liljenberg - magnus.liljenberg@astrazeneca.com; Tore Brinck- tore@physchem.kth.se

*Corresponding author

Coordinates of all optimized structures, electronic energies, *SS* values calculated with larger basis set and zero point energies.

Contents

Coordinates (in xyz-format) for entries 1–12, Table 1 2
Coordinates (in xyz-format) for entries 1–8, Table 2 8
Coordinates (in xyz-format) for entries 1–13, Table 3 14
In vacuo Energies for entries 1–12, Table 1........ 21
In vacuo Energies for entries 1–8, Table 2........ 22
In vacuo Energies for entries 1–13, Table 3........ 23
Sigma Stability values for reaction series A calculated with a larger basis set 24
Zero point energies for entries 1–11, Table 1........ 24

Coordinates (in xyz-format) for entries 1–12, Table 1

11
Table 1 entry 1 starting material

C1	2.690781	-2.611010	0.017279
N2	1.565374	-1.928969	0.012945
C3	1.629622	-0.615049	0.009090
C4	2.823865	0.095769	0.009785
C5	4.019064	-0.624568	0.014657
C6	3.947568	-2.018531	0.018354
Cl7	5.538821	0.182732	0.016057
F8	2.822249	1.429289	0.005930
F9	2.607309	-3.941744	0.020726
F10	0.480156	0.060782	0.004363
F11	5.052624	-2.765212	0.022806

15
Table 1 entry 1 sigma-complex

C1	-2.026805	1.513136	-0.083747
C2	-0.786023	2.128619	-0.369297
C3	0.315793	1.310641	-0.328902
N4	0.313484	0.014457	-0.066359
C5	-0.876412	-0.656020	0.048354
C6	-2.072957	0.171190	0.171519
F8	1.530719	1.863850	-0.533906
Cl8	-3.476015	2.477055	-0.052029
F12	-1.066492	-1.617292	-1.061211
N12	-0.774987	-1.726555	1.165406
H13	-0.618791	-1.299834	2.082774
H14	0.018653	-2.341923	0.959917
H15	-1.619415	-2.305036	1.206435
F14	-0.700012	3.456179	-0.633470
F15	-3.237851	-0.462798	0.464880

11
Table 1 entry 2 starting material

C1	2.689688	-2.600943	0.017036
N2	1.558714	-1.928344	0.012986
C3	1.642495	-0.608772	0.009084
C4	2.820713	0.125436	0.009552
C5	4.004736	-0.613673	0.014559
C6	3.947804	-2.006972	0.018265
F7	5.051333	-2.759444	0.022855
Cl8	5.550806	0.169441	0.016212
H9	2.812041	1.208159	0.006058
F10	2.612211	-3.932165	0.020439
F11	0.474862	0.042906	0.004603

15
Table 1 entry 2 sigma-complex

C1	-2.007284	1.524289	-0.064552
C2	-0.772199	2.167235	-0.330615

C3	0.310135	1.316071	-0.289574
N4	0.321258	0.015307	-0.053525
C5	-0.875669	-0.649693	0.059287
C6	-2.072210	0.183535	0.168800
F7	1.541263	1.850219	-0.481417
Cl8	-3.482634	2.471311	-0.030603
F9	-1.058751	-1.608594	-1.038044
N10	-0.786403	-1.704260	1.193240
H11	-0.616362	-1.263264	2.101368
H12	-0.005809	-2.339053	0.997609
H13	-1.641799	-2.264422	1.251426
H14	-0.681639	3.228387	-0.517223
F15	-3.241339	-0.459757	0.436329

10

Table 1 entry 3 starting material

C1	2.675334	-2.615783	0.017190
N2	1.546133	-1.941020	0.012756
C3	1.619545	-0.627715	0.009123
C4	2.852768	0.027177	0.010193
N5	3.981891	-0.647533	0.014882
C6	3.908448	-1.960934	0.018193
F7	5.039224	-2.659440	0.022572
F9	2.907484	1.355201	0.006323
F10	2.620499	-3.943793	0.020805
F11	0.488885	0.070940	0.004446

14

Table 1 entry 3 sigma-complex

C1	-1.842205	1.508450	0.157053
C2	-0.756359	2.024727	-0.500594
N3	0.315970	1.246452	-0.709182
C4	0.258302	0.017576	-0.332542
C5	-0.943001	-0.623962	0.217477
N6	-1.924213	0.255533	0.586446
F7	-1.427079	-1.624664	-0.754229
N8	-0.637874	-1.614359	1.355658
H9	0.045890	-2.318584	1.062258
F10	1.347564	-0.775114	-0.493717
F11	-0.689728	3.316658	-0.910522
H12	-1.503172	-2.100755	1.613418
H13	-0.284341	-1.133367	2.187819
F14	-2.888736	2.306351	0.450096

11

Table 1 entry 4 starting material

C1	1.065205	3.966074	4.675460
C2	0.230655	3.423245	3.704831
C3	0.479225	3.773703	2.380371
C4	1.533359	4.636814	2.085791
C5	2.290801	5.107832	3.150760
N6	2.062594	4.781894	4.406956
F7	-0.279752	3.291126	1.404445
F8	0.852576	3.649167	5.953454
F9	-0.770625	2.598771	4.018300

F10	1.792168	4.987670	0.824006
F11	3.307151	5.935301	2.903364

15

Table 1 entry 4 sigma-complex

C1	-1.897057	1.527900	0.067336
N2	-0.760836	2.204329	-0.082473
C3	0.329249	1.440833	-0.077019
C4	0.355552	0.082963	0.068251
C5	-0.860426	-0.702686	0.147112
C6	-2.011215	0.175314	0.221453
F8	1.504069	2.090631	-0.212280
F11	-3.025416	2.267791	0.080936
F12	-0.969162	-1.667522	-0.941286
N12	-0.823967	-1.747666	1.296975
H13	-0.743180	-1.295019	2.211903
H14	-0.027791	-2.380422	1.173178
H15	-1.675392	-2.317358	1.287646
F14	1.530537	-0.612544	0.071713
F15	-3.227263	-0.424942	0.378615

11

Table 1 entry 5 starting material

C1	2.691655	-2.613678	0.017321
N2	1.565648	-1.931113	0.013137
C3	1.629256	-0.616153	0.009348
C4	2.816516	0.113623	0.009854
C5	3.997532	-0.632236	0.014898
C6	3.947345	-2.022837	0.018503
F7	5.068002	-2.747107	0.022865
F8	5.177741	-0.028003	0.016038
Cl9	2.846551	1.841340	0.004484
F10	2.606183	-3.942511	0.020495
F11	0.468591	0.038129	0.004575

15

Table 1 entry 5 sigma-complex

C1	-1.946699	1.535814	0.083443
N2	-0.808991	2.225325	-0.024428
C3	0.283652	1.475065	-0.007354
C4	0.337180	0.107511	0.127457
C5	-0.885826	-0.688376	0.175017
C6	-2.050124	0.185684	0.213882
F8	1.441732	2.151999	-0.121644
F11	-3.077610	2.268124	0.072999
F12	-0.974523	-1.642737	-0.903661
N12	-0.917612	-1.715003	1.341451
H13	-0.810412	-1.248987	2.246998
H14	-0.170409	-2.409392	1.240911
H15	-1.809253	-2.220104	1.340650
Cl14	1.872058	-0.749585	0.069147
F15	-3.261422	-0.432056	0.325617

10

Table 1 entry 6 starting material

C1	1.068080	3.970436	4.655075
C2	0.212718	3.410749	3.688162
C3	0.455512	3.755040	2.378919
C4	1.533701	4.629324	2.149609
N5	2.293733	5.116353	3.094169
N6	2.055056	4.779322	4.375938
F7	-0.280044	3.293631	1.381448
F8	0.867943	3.664388	5.930439
F9	-0.772233	2.596220	4.030161
F10	1.803825	4.988017	0.900692

14

Table 1 entry 6 sigma-complex

C1	-1.972740	1.389336	0.099594
N2	-0.887512	2.146914	0.205292
N3	0.276925	1.467998	0.383453
C4	0.300489	0.182742	0.428949
C5	-0.808654	-0.755636	0.246499
C6	-2.017562	0.021039	0.155426
F11	-3.124968	2.063694	-0.054739
F12	-0.577012	-1.616690	-0.891232
N12	-0.841206	-1.852563	1.330383
H13	0.038546	-2.378706	1.332386
H14	-1.599357	-2.514749	1.137553
H15	-0.987443	-1.453465	2.262622
F18	-3.195700	-0.653083	0.062013
F14	1.503365	-0.412269	0.628203

14

Table 1 entry 7 starting material

C1	1.064300	3.964468	4.681431
C2	0.231856	3.423558	3.709070
C3	0.491580	3.781314	2.393591
C4	1.544961	4.647110	2.074578
C5	2.289610	5.106135	3.160505
N6	2.061820	4.779744	4.419604
F7	-0.279690	3.286669	1.432344
F8	0.848223	3.645509	5.953024
F9	-0.768894	2.600035	4.021063
F10	3.313157	5.934504	2.967593
C11	1.788978	5.007868	0.629514
F12	2.828146	5.841213	0.470835
F13	2.035703	3.902559	-0.106001
F14	0.701966	5.603557	0.093642

18

Table 1 entry 7 sigma-complex

C1	-1.979735	1.241543	-0.504956
C2	-0.908863	2.122725	-0.411748
C3	0.280518	1.502054	0.072916
C4	0.339152	0.176256	0.351891
C5	-0.798921	-0.691796	0.056110
N6	-1.982418	-0.045620	-0.244320
F11	-3.185800	1.720671	-0.870482

F12	-0.439770	-1.627276	-0.991323
N12	-1.051216	-1.732935	1.169521
H13	-0.206192	-2.275932	1.371633
H14	-1.776904	-2.386169	0.856003
H15	-1.374189	-1.290146	2.034380
F15	1.373143	2.261888	0.268842
C18	-0.947898	3.581749	-0.682262
F19	0.078533	3.984180	-1.481948
F20	-2.086839	3.979935	-1.286990
F21	-0.833034	4.335103	0.450598
F18	1.475712	-0.410385	0.816086

18

Table 1 entry 8 sigma-complex (the starting material for this entry is the same as for entry 7)

C1	-1.955629	1.445767	0.168643
N2	-0.823564	2.165484	0.137657
C3	0.271590	1.437880	0.197388
C4	0.385470	0.062530	0.324924
C5	-0.840192	-0.749922	0.313493
C6	-2.030871	0.096973	0.271281
F8	1.418884	2.145660	0.128839
F11	-3.095593	2.156616	0.100600
F12	-0.855497	-1.707346	-0.755210
N12	-0.959540	-1.753672	1.497372
H13	-0.984146	-1.261840	2.394827
H14	-0.167909	-2.402933	1.503511
H15	-1.818893	-2.305325	1.408724
C15	1.727865	-0.572485	0.230290
F15	1.661221	-1.917327	0.459380
F16	2.311408	-0.430475	-0.989005
F17	2.622511	-0.075013	1.119940
F18	-3.226784	-0.555020	0.301399

17

Table 1 entry 9 starting material

C1	2.736800	-2.580715	0.018235
N2	1.600187	-1.917132	0.014924
C3	1.681289	-0.603559	0.011069
C4	2.862341	0.139962	0.010385
C5	4.042744	-0.613084	0.014247
C6	4.011649	-2.013021	0.018621
F7	5.211254	0.010758	0.013584
F8	2.632646	-3.906188	0.021537
F9	0.522088	0.047511	0.007528
C10	5.222463	-2.916291	0.023434
F11	6.379110	-2.237847	0.027351
F12	5.216061	-3.718710	1.109024
F13	5.223981	-3.719705	-1.061211
C14	2.785677	1.648587	0.004794
F15	3.992982	2.232346	0.009997
F16	2.123527	2.089583	-1.085615
F17	2.110911	2.096977	1.084311

21

Table 1 entry 9 sigma-complex

C1	-1.931578	1.383135	-0.207962
C2	-0.782539	2.185201	-0.244878
C3	0.401946	1.444171	-0.072667
C4	0.430200	0.088709	0.176124
C5	-0.853276	-0.648081	0.160151
N6	-2.017502	0.105284	-0.002387
F8	1.578059	2.083363	-0.169179
F11	-3.110626	2.006053	-0.401289
F12	-0.808198	-1.674375	-0.829550
N12	-1.079875	-1.543552	1.410104
H13	-1.212315	-0.972984	2.249706
H14	-0.294671	-2.181283	1.566172
H15	-1.928054	-2.103693	1.271886
C15	-0.885303	3.651546	-0.477459
F15	0.290964	4.293922	-0.317501
F16	-1.326829	3.958166	-1.729176
F17	-1.770475	4.243292	0.369147
C18	1.727701	-0.643779	0.176697
F19	1.579095	-1.928034	0.615113
F20	2.299158	-0.740191	-1.052814
F21	2.663975	-0.073813	0.973074

10

Table 1 entry 10 starting material

C1	2.723940	-2.545023	0.017142
N2	1.554668	-1.929105	0.012697
C3	1.614924	-0.609558	0.009071
C4	2.811687	0.098096	0.009807
C5	3.958893	-0.686983	0.014623
N6	3.930995	-2.007712	0.018633
F8	5.143628	-0.099850	0.015645
F9	2.855320	1.428949	0.006123
F10	2.680088	-3.868812	0.020548
F11	0.472021	0.055476	0.004515

14

Table 1 entry 10 sigma-complex

C1	-1.928733	1.512392	0.045882
N2	-0.752915	2.136638	-0.171144
C3	0.262304	1.296134	-0.191906
N4	0.310737	-0.005215	-0.049453
C5	-0.897996	-0.682252	0.061366
C6	-2.073398	0.178316	0.207222
F8	1.461201	1.879766	-0.372970
F11	-3.003924	2.313713	0.105075
F12	-1.080623	-1.583454	-1.038712
N12	-0.790601	-1.728163	1.188568
H13	-0.582148	-1.283782	2.087235
H14	-0.028907	-2.379291	0.970447
H15	-1.655235	-2.269437	1.280852
F14	-3.277617	-0.411578	0.430010

21

Table 1 entry 11 sigma-complex (the starting material for this entry is the same as for entry 9)

C1	-1.892700	1.491384	0.333497
N2	-0.764497	2.182059	0.240184
C3	0.323116	1.424592	0.196469
C4	0.409191	0.057686	0.305593
C5	-0.839670	-0.728064	0.443548
C6	-2.046720	0.131726	0.457313
F8	1.463982	2.114133	0.017695
F11	-3.003282	2.249241	0.294203
F12	-0.932064	-1.723599	-0.564776
N12	-0.790293	-1.652557	1.698072
H13	-0.716980	-1.094385	2.553381
H14	0.016749	-2.281685	1.651339
H15	-1.636837	-2.226270	1.757011
C15	1.721955	-0.613379	0.078079
F15	1.663740	-1.945701	0.368639
F16	2.152757	-0.529664	-1.206353
F17	2.720778	-0.099894	0.835255
C18	-3.415020	-0.459068	0.391974
F19	-3.402717	-1.792733	0.683585
F20	-4.284274	0.113942	1.258245
F21	-3.989338	-0.349944	-0.833007

15
Table 1 entry 12 sigma-complex

C1	0.471796	2.061718	0.080562
C2	-0.275792	3.221002	0.143642
N3	-1.595262	3.180996	0.055703
C4	-2.193350	2.035453	-0.085950
C5	-1.603473	0.712986	-0.0721270
C6	-0.167891	0.833245	-0.085443
F7	1.813781	2.116652	0.153901
F8	0.320311	4.430727	0.287631
Cl9	0.795514	-0.631942	-0.137699
F11	-2.124417	-0.106951	1.068647
N12	-2.210591	-0.218536	-1.169875
H13	-1.887474	-1.183042	-1.048570
H14	-1.962431	0.097993	-2.111516
H15	-3.231542	-0.224490	-1.086128
F15	-3.555085	2.045129	-0.192173

Coordinates (in xyz-format) for entries 1–8, Table 2

15
Table 2 entry 1 starting material

C1	2.757033	-2.719896	0.113556
C2	1.544948	-2.029961	0.059854
C3	1.610882	-0.639353	-0.038128
C4	2.822137	0.044526	-0.049391
C5	4.012167	-0.673818	0.021869
C6	3.982970	-2.063053	0.104099
F7	5.128834	-2.752698	0.171344
F8	5.182404	-0.029994	0.013027

F9	2.851904	1.380882	-0.129956
F10	2.759635	-4.060039	0.190142
F11	0.479982	0.080070	-0.112071
C12	0.229978	-2.757486	0.039645
H13	-0.563121	-2.132302	0.454987
F14	-0.113597	-3.077259	-1.283666
H15	0.295506	-3.688297	0.607038

19

Table 2 entry 1 sigma-complex

C1	-1.848943	1.552376	-0.007084
C2	-0.680674	2.199425	-0.440760
C3	0.441291	1.357202	-0.503549
C4	0.405187	0.015877	-0.203916
C5	-0.808802	-0.695651	0.108930
C6	-1.911452	0.213036	0.297343
F8	1.633444	1.883500	-0.872268
F11	-2.984948	2.277456	0.128386
F12	-1.120863	-1.752157	-0.911337
N12	-0.639846	-1.706286	1.277006
H13	-0.413567	-1.235162	2.157533
H14	0.107804	-2.372338	1.061881
H15	-1.501609	-2.244349	1.406045
F14	1.540176	-0.744785	-0.291130
F15	-3.088868	-0.348376	0.712447
C16	-0.614314	3.656866	-0.705784
F17	-0.301692	4.398750	0.470526
H18	0.164038	3.891922	-1.436271
H19	-1.572842	4.038521	-1.066747

18

Table 2 entry 2 starting material

C1	1.019027	0.920757	0.000000
C2	-0.295339	1.388924	0.000000
C3	-1.317995	0.438781	0.000000
C4	-1.018964	-0.920848	0.000000
C5	0.295329	-1.388945	0.000000
C6	1.318297	-0.438859	0.000000
F7	2.607325	-0.787328	0.000000
F9	-2.035435	-1.790540	0.000000
F10	2.035262	1.790752	0.000000
F11	-2.606884	0.786987	0.000000
C12	-0.525218	2.885563	0.000000
F13	-1.825837	3.211395	0.000000
F14	0.038280	3.454442	1.085969
F15	0.038280	3.454442	-1.085969
C15	0.525220	-2.885447	0.000000
F16	1.825734	-3.211598	0.000000
F17	-0.038475	-3.454252	1.085678
F18	-0.038475	-3.454252	-1.085678

22

Table 2 entry 2 sigma-complex

C1	-2.054128	1.462652	-0.024685

C2	-0.815814	2.140861	-0.108046
C3	0.347316	1.418684	-0.005099
C4	0.382979	0.031702	0.218594
C5	-0.851621	-0.731778	0.276505
C6	-2.047427	0.107969	0.196957
F8	1.521909	2.075805	-0.118733
F12	-0.922786	-1.785639	-0.733846
N12	-0.972399	-1.704027	1.516795
H13	-0.983536	-1.181323	2.396575
H14	-0.181256	-2.352666	1.535267
H15	-1.831812	-2.257820	1.454200
F15	-3.199618	-0.608453	0.304915
F14	-0.780935	3.488392	-0.286350
C16	1.701911	-0.655887	0.153214
F16	1.600461	-1.985254	0.447151
F17	2.289912	-0.593911	-1.072637
F18	2.619268	-0.145889	1.016299
C19	-3.317186	2.282168	-0.151958
F20	-4.439552	1.543320	-0.210532
F21	-3.456717	3.131013	0.893787
F22	-3.290392	3.044506	-1.269371

15

Table 2 entry 3 starting material

C1	2.751809	-2.704362	0.132170
C2	1.551034	-2.020917	-0.049669
C3	1.609301	-0.634454	-0.213002
C4	2.819376	0.048649	-0.192638
C5	4.005634	-0.659646	-0.009987
C6	3.975135	-2.040271	0.155481
F7	5.113331	-2.720697	0.331560
F9	2.855471	1.376729	-0.349419
F10	2.752045	-4.036273	0.294658
F11	0.483896	0.069259	-0.388625
C12	0.237696	-2.754762	-0.071229
F13	-0.576788	-2.284142	0.926903
F14	-0.409771	-2.516034	-1.255497
H14	0.353253	-3.833633	0.051936
F15	5.170881	-0.010109	0.010533

19

Table 2 entry 3 sigma-complex

C1	-1.986329	1.484382	-0.096320
C2	-0.791407	2.196736	-0.306098
C3	0.370768	1.414834	-0.215625
C4	0.357966	0.071476	0.070621
C5	-0.851288	-0.709395	0.175157
C6	-2.019539	0.142075	0.188103
F7	1.576377	2.007226	-0.385242
F8	-0.937446	-1.754484	-0.877051
N9	-0.821782	-1.714097	1.361638
H10	-0.760195	-1.233552	2.263706
H11	-0.017970	-2.342558	1.270586
H12	-1.666183	-2.294013	1.357216
F13	1.531699	-0.626686	0.156328

F14	-3.213937	-0.491400	0.393672
F15	-3.162891	2.146082	-0.145038
C16	-0.744849	3.643919	-0.606393
H17	0.276365	4.011070	-0.735040
F18	-1.454706	3.959174	-1.754954
F19	-1.342733	4.397972	0.391745

14

Table 2 entry 4 starting material

C1	2.709086	-2.697123	0.017421
C2	1.523960	-1.947314	0.012410
C3	1.571937	-0.560834	0.008770
C4	2.805729	0.106256	0.009703
C5	3.990093	-0.642840	0.014415
C6	3.942339	-2.030763	0.018305
F7	5.075306	-2.728644	0.023036
F15	5.168339	-0.024077	0.015399
F11	0.346929	-2.567278	0.011251
F10	0.439870	0.138136	0.004410
N11	2.891845	2.692131	0.002973
C13	2.852904	1.531239	0.005984
N13	2.612593	-5.282059	0.025029
C15	2.657786	-4.121256	0.021631

18

Table 2 entry 4 sigma-complex

C1	-1.991689	1.478252	-0.062541
C2	-0.757110	2.167319	-0.187606
C3	0.397783	1.439870	-0.096645
C4	0.405883	0.043587	0.128704
C5	-0.832240	-0.737696	0.187073
C6	-2.014009	0.119685	0.153332
F8	1.583393	2.061338	-0.215940
F12	-0.906237	-1.737197	-0.862187
N12	-0.897312	-1.733942	1.382975
H13	-0.813209	-1.253133	2.283662
H14	-0.147278	-2.430167	1.313237
H15	-1.784393	-2.249085	1.371942
F15	-3.197690	-0.525939	0.278959
F16	-0.748106	3.503991	-0.392755
N15	-4.207341	2.807206	-0.232375
C17	-3.216846	2.204328	-0.153293
N17	2.652701	-1.222055	0.176369
C19	1.641540	-0.637061	0.148045

18

Table 2 entry 5 starting material

C1	2.691445	-2.658027	0.018627
C2	1.501977	-1.929882	0.013033
C3	1.560453	-0.512461	0.009180
C4	2.822758	0.089814	0.009012
C5	3.999426	-0.660605	0.013786
C6	3.938766	-2.041506	0.018986
F7	5.051800	-2.770532	0.023914
F8	2.997133	1.415337	0.004260

F9	2.671916	-3.995859	0.023524
C10	0.249315	-2.800843	0.011016
F11	-0.908241	-2.127612	0.003566
F12	0.245150	-3.606161	-1.073697
F13	0.235778	-3.597957	1.101370
F14	5.177237	-0.039581	0.013182
C15	0.307747	0.357353	0.004850
F16	0.585798	1.670929	0.002622
F17	-0.446434	0.123044	-1.088991
F18	-0.451458	0.127877	1.096373

22
Table 2 entry 5 sigma-complex

C1	-2.021212	1.518711	-0.093790
C2	-0.793549	2.198889	-0.368949
C3	0.350011	1.381380	-0.387038
C4	0.331924	0.038218	-0.097852
C5	-0.860557	-0.702916	0.195226
C6	-2.012004	0.179055	0.232402
F8	1.560113	1.891808	-0.696586
F12	-1.077309	-1.813238	-0.745229
N12	-0.737193	-1.608228	1.462692
H13	-0.570023	-1.055937	2.308529
H14	0.037336	-2.269364	1.349640
H15	-1.592527	-2.156551	1.593865
F14	1.493179	-0.676338	-0.128524
F15	-3.153195	-0.486622	0.576397
C16	-3.360329	2.232414	-0.153955
F16	-4.382339	1.400875	-0.446748
F17	-3.674705	2.834939	1.016238
F18	-3.377677	3.183129	-1.114975
C19	-0.672713	3.674816	-0.575589
F20	0.565538	4.137407	-0.278285
F21	-0.918757	4.085507	-1.852371
F22	-1.526631	4.378265	0.214965

13
Table 2 entry 6 starting material

C1	2.738850	-2.689790	0.017512
C2	1.534640	-1.971522	0.012750
C3	1.589605	-0.570739	0.008854
C4	2.806509	0.094680	0.009811
C5	3.991106	-0.639191	0.014688
C6	3.960166	-2.032558	0.018475
F7	5.100384	-2.725265	0.022950
F8	5.159727	-0.005518	0.015680
F9	2.846922	1.428229	0.006025
F10	2.716687	-4.022993	0.021214
F11	0.460476	0.137982	0.004178
N12	-0.736477	-3.204402	0.011038
C13	0.284693	-2.649638	0.011838

17
Table 2 entry 6 sigma complex

C1	-1.987144	1.491034	-0.077518

C2	-0.771709	2.196192	-0.259377
C3	0.401793	1.406986	-0.171087
C4	0.370640	0.066709	0.083777
C5	-0.857123	-0.709638	0.168415
C6	-2.030655	0.150474	0.174622
F8	1.595064	2.011392	-0.319168
F11	-3.142960	2.178047	-0.132608
F12	-0.932670	-1.713909	-0.884672
N12	-0.845823	-1.699001	1.357791
H13	-0.799121	-1.210768	2.257134
H14	-0.039103	-2.328149	1.287662
H15	-1.690371	-2.279800	1.345042
F14	1.526514	-0.650428	0.173132
F15	-3.223952	-0.483542	0.354298
N16	-0.700840	4.724081	-0.741143
C17	-0.733188	3.575665	-0.523936

14
Table 2 entry 7 starting material

C1	2.721619	-2.709361	0.017554
C2	1.517632	-1.959516	0.012632
C3	1.583121	-0.568116	0.008896
C4	2.811208	0.090309	0.009835
C5	3.991048	-0.644499	0.014595
C6	3.941822	-2.037222	0.018435
F7	5.084245	-2.719492	0.023024
F8	5.162217	-0.021188	0.015481
F9	2.853646	1.416356	0.006132
F11	0.467453	0.156477	0.004370
N11	-0.798253	-3.113120	0.010476
C13	0.243113	-2.597796	0.011431
N13	2.704753	-5.296584	0.024882
C15	2.710204	-4.134700	0.021602

18
Table 2 entry 7 sigma complex

C1	-2.005186	1.503587	-0.076864
C2	-0.770472	2.198035	-0.272168
C3	0.393346	1.407705	-0.181026
C4	0.362188	0.062904	0.072063
C5	-0.856635	-0.719348	0.166439
C6	-2.026628	0.151226	0.167920
F8	1.590193	2.000036	-0.330207
F12	-0.948872	-1.715350	-0.886359
N12	-0.841469	-1.705430	1.355156
H13	-0.781171	-1.221878	2.256816
H14	-0.040158	-2.340587	1.276097
H15	-1.688758	-2.283250	1.351291
F14	1.521601	-0.639472	0.167410
F15	-3.207047	-0.487613	0.365223
N15	-0.633222	4.730276	-0.728488
C17	-0.697633	3.582058	-0.521466
N17	-4.251149	2.792422	-0.180669
C19	-3.246236	2.209426	-0.132063

Table 2 entry 8 is the same as Table 1 entry 4, only the temperature and thus the experimental rate constants are different.

Coordinates (in xyz-format) for entries 1–13, Table 3

```
    12
Table 3 entry 1 starting material
 C17      0.000000    1.194218   -0.696663
 C18      0.000000    1.194218    0.696663
 C19      0.000000    0.000000    1.406348
 C20      0.000000   -1.194218    0.696663
 C21      0.000000   -1.194218   -0.696663
 C22      0.000000    0.000000   -1.406348
 F7       0.000000    2.369877   -1.352108
 F8       0.000000    2.369877    1.352108
 H9       0.000000    0.000000    2.492150
 F10      0.000000   -2.369877   -1.352108
 H11      0.000000    0.000000   -2.492150
 F12      0.000000   -2.369877    1.352108

    17
Table 3 entry 1 sigma-complex
 C1      -0.712940    1.231592   -0.489781
 C2      -1.894786    2.045837   -0.291144
 C3      -3.062160    1.241251    0.001200
 C4      -2.952714   -0.103608    0.317718
 C5      -1.756295   -0.800374    0.237218
 C6      -0.616730   -0.105898   -0.185614
 H7      -4.039244    1.707322    0.066267
 F8      -1.695404   -2.135619    0.549519
 F9      -1.596799    3.008533    0.995258
 O10     -1.998279    3.045097   -1.282672
 C11     -3.052319    3.987020   -1.118610
 H12     -2.898663    4.767299   -1.869614
 H13     -4.039173    3.534482   -1.288397
 H14     -3.021257    4.424630   -0.117741
 H15      0.334084   -0.619577   -0.298516
 F16     -4.081121   -0.789237    0.670889
 F17      0.409443    1.887197   -0.936537

    12
Table 3 entry 2 starting material
 C17      3.266860   -1.609794   -0.089956
 C18      2.163657   -2.351187    0.319141
 C19      0.900372   -1.763166    0.316467
 C20      0.727389   -0.446206   -0.088950
 C21      1.836589    0.299228   -0.499908
 C22      3.094931   -0.288308   -0.497120
 F7       4.484786   -2.173528   -0.088587
 F8       2.326187   -3.624135    0.712047
 F9      -0.147588   -2.506589    0.717903
 H10      1.731846    1.330843   -0.821423
 F11      4.181946    0.403111   -0.887501
```

H12	-0.268273	-0.013609	-0.080579

17

Table 3 entry 2 sigma-complex

C1	-0.779595	1.283502	-0.440205
C2	-1.998176	2.047794	-0.282560
C3	-3.101432	1.136792	-0.008235
C4	-2.993207	-0.206049	0.258040
C5	-1.747643	-0.866227	0.194435
C6	-0.666200	-0.063953	-0.160645
F7	-4.355724	1.720737	0.034127
H8	-1.623858	-1.924009	0.391985
F10	-1.853025	3.033144	0.913805
O11	-2.171448	2.963082	-1.360747
C12	-2.983109	4.119960	-1.148379
H13	-3.071101	4.606273	-2.124907
H14	-3.982239	3.859744	-0.789423
H15	-2.519912	4.811885	-0.440322
F15	0.575796	-0.622645	-0.279561
H16	-3.903526	-0.753647	0.491763
F17	0.348122	1.978756	-0.807498

12

Table 3 entry 3 starting material

C17	3.266972	-1.611540	-0.089420
C18	2.153839	-2.342049	0.317476
C19	0.880400	-1.786449	0.327166
C20	0.757158	-0.464173	-0.087523
C21	1.837279	0.307979	-0.503105
C22	3.090628	-0.291529	-0.495349
F7	4.488720	-2.170130	-0.090268
F8	2.337740	-3.614450	0.706622
H9	0.021736	-2.366815	0.646908
H10	1.713892	1.337412	-0.821545
F11	4.172708	0.402123	-0.885340
F12	-0.468742	0.096378	-0.086262

17

Table 3 entry 3 sigma-complex

C1	-0.711565	1.269426	-0.410412
C2	-1.900318	2.089866	-0.206079
C3	-3.078838	1.271179	0.083928
C4	-2.974274	-0.087351	0.284844
C5	-1.805950	-0.841546	0.167232
C6	-0.680653	-0.082654	-0.192325
H7	-4.043618	1.750212	0.205331
H8	-1.760449	-1.908559	0.340201
F10	-1.645616	3.028456	0.980349
O11	-2.007844	3.038554	-1.265423
C12	-3.079431	3.968875	-1.167451
H13	-2.956351	4.679957	-1.989504
H14	-4.058692	3.482123	-1.275734
H15	-3.050533	4.505976	-0.214763
F15	0.511474	-0.725192	-0.358571
F16	-4.122963	-0.762688	0.610620

F17	0.441603	1.917543	-0.783166

12

Table 3 entry 4 starting material

C17	3.257665	-1.617397	-0.086089
C18	2.168197	-2.374995	0.326213
C19	0.912961	-1.778996	0.319827
C20	0.737109	-0.458583	-0.086191
C21	1.842934	0.281511	-0.495079
C22	3.109961	-0.295034	-0.496997
F7	4.488889	-2.152925	-0.096134
H8	2.294396	-3.404458	0.644828
F9	-0.167619	-2.473871	0.710084
F10	1.687431	1.551231	-0.887488
F11	4.170107	0.423887	-0.892292
F12	-0.479896	0.103500	-0.086933

17

Table 3 entry 4 sigma-complex

C1	-0.702686	1.295385	-0.412257
C2	-1.939796	2.043396	-0.237392
C3	-3.067247	1.156219	0.019991
C4	-2.935332	-0.183674	0.295610
C5	-1.696775	-0.834671	0.236342
C6	-0.607081	-0.053156	-0.129030
F7	-4.332915	1.695932	0.048485
F8	-1.585262	-2.165913	0.529797
F10	-1.817263	3.008118	0.968988
O11	-2.103958	2.970897	-1.308879
C12	-2.949622	4.107157	-1.105104
H13	-3.003665	4.616985	-2.071692
H14	-3.958123	3.818856	-0.797897
H15	-2.530607	4.787253	-0.359178
F15	0.603495	-0.669712	-0.244870
F16	-4.040243	-0.912153	0.598720
H17	0.176949	1.833585	-0.748665

12

Table 3 entry 5 starting material

C17	3.269934	-1.611265	-0.090017
C18	2.165510	-2.352486	0.319234
C19	0.899612	-1.774483	0.320389
C20	0.739510	-0.453589	-0.088295
C21	1.840758	0.298914	-0.500462
C22	3.106340	-0.290500	-0.497977
F7	4.485634	-2.169484	-0.090378
F8	2.320477	-3.619998	0.711364
F9	-0.160200	-2.489505	0.714267
Cl10	1.639310	1.944936	-1.009064
F11	4.177838	0.404826	-0.887332
F12	-0.483190	0.083348	-0.080308

17

Table 3 entry 5 sigma-complex

C1	-0.742476	1.309835	-0.421370

C2	-1.969327	2.070108	-0.253247
C3	-3.077074	1.164832	-0.008018
C4	-2.942574	-0.175214	0.287836
C5	-1.704539	-0.813698	0.236552
C6	-0.603119	-0.035228	-0.126473
F7	-4.343676	1.693185	-0.017897
F8	-1.583973	-2.125411	0.584212
F10	-1.817790	3.033640	0.960500
O11	-2.137794	2.991393	-1.318477
C12	-2.949591	4.148219	-1.092506
H13	-3.033354	4.644314	-2.063700
H14	-3.949821	3.883004	-0.740476
H15	-2.486353	4.828022	-0.373991
Cl15	0.978380	-0.792120	-0.236370
F16	-4.047166	-0.901011	0.592338
F17	0.351215	2.017587	-0.831694

12

Table 3 entry 6 starting material

C17	0.696271	1.205977	0.000000
C18	1.392543	0.000000	0.000000
C19	0.696271	-1.205977	0.000000
C20	-0.696271	-1.205977	0.000000
C21	-1.392543	0.000000	0.000000
C22	-0.696271	1.205977	0.000000
F7	1.365224	2.364637	0.000000
F8	2.730448	0.000000	0.000000
F9	1.365224	-2.364637	0.000000
F10	-2.730448	0.000000	0.000000
F11	-1.365224	2.364637	0.000000
F12	-1.365224	-2.364637	0.000000

17

Table 3 entry 6 sigma-complex

C1	-0.745441	1.289458	-0.438493
C2	-1.965522	2.053762	-0.266653
C3	-3.076962	1.152431	-0.013897
C4	-2.946664	-0.189075	0.278526
C5	-1.711930	-0.834988	0.214173
C6	-0.615099	-0.053306	-0.145169
F7	-4.342938	1.687686	-0.008658
F8	-1.575184	-2.150140	0.554096
F10	-1.809580	3.022802	0.948805
O11	-2.132509	2.978527	-1.330589
C12	-2.954452	4.127851	-1.106275
H13	-3.036709	4.627380	-2.075954
H14	-3.954479	3.854153	-0.760359
H15	-2.500454	4.808691	-0.382771
F15	0.606876	-0.635003	-0.240960
F16	-4.052918	-0.911073	0.588365
F17	0.371144	1.974377	-0.839495

17

Table 3 entry 7 sigma-complex (the starting material for this entry is the same as for entry 4)

C1	-0.757578	1.271425	-0.439096

C2	-1.981463	2.038641	-0.273099
C3	-3.094870	1.136137	-0.010695
C4	-2.953234	-0.205508	0.254777
C5	-1.729426	-0.880913	0.194109
C6	-0.647025	-0.072284	-0.165184
F7	-4.355826	1.689358	0.035227
H8	-1.629176	-1.936095	0.410845
F10	-1.827412	3.002773	0.916828
O11	-2.148892	2.950498	-1.352565
C12	-2.971102	4.101581	-1.144197
H13	-3.042906	4.596454	-2.117229
H14	-3.975596	3.832912	-0.806356
H15	-2.522716	4.787736	-0.421048
F15	0.586478	-0.641230	-0.283027
F16	-4.079487	-0.913225	0.555455
F17	0.363480	1.971782	-0.809241

17

Table 3 entry 8 sigma-complex (the starting material for this entry is the same as for entry 5)

C1	-0.742503	1.306011	-0.461058
C2	-1.974559	2.068864	-0.279313
C3	-3.087751	1.156348	-0.011298
C4	-2.954551	-0.181277	0.261595
C5	-1.711706	-0.824681	0.193988
C6	-0.621688	-0.047519	-0.169681
F7	-4.347951	1.698380	-0.002468
F8	-1.581470	-2.144786	0.515869
F10	-1.835521	3.023055	0.898450
O11	-2.195801	2.959907	-1.362508
C12	-2.969685	4.140226	-1.129285
H13	-3.109798	4.599006	-2.112432
H14	-3.949006	3.911080	-0.701086
H15	-2.447060	4.841275	-0.474054
F15	0.589170	-0.647314	-0.258401
F16	-4.053799	-0.915486	0.560756
Cl17	0.702895	2.190634	-0.957936

17

Table 3 entry 9 sigma-complex (the starting material for this entry is the same as for entry 5)

C1	-0.723545	1.311499	-0.407177
C2	-1.948321	2.080394	-0.234214
C3	-3.060945	1.172124	0.025974
C4	-2.929846	-0.169964	0.289487
C5	-1.694147	-0.829225	0.217292
C6	-0.598469	-0.031134	-0.138707
F7	-4.322386	1.716597	0.058039
Cl8	-1.520917	-2.527562	0.609357
F10	-1.794483	3.032275	0.946256
O11	-2.119035	2.980782	-1.319155
C12	-2.946203	4.131804	-1.119270
H13	-3.024375	4.614617	-2.097488
H14	-3.947249	3.861119	-0.773896
H15	-2.497616	4.827868	-0.406279
F15	0.625913	-0.599584	-0.253056
F16	-4.043771	-0.881540	0.587750

F17	0.392106	2.007707	-0.789360

12

Table 3 entry 10 starting material

C17	-0.149526	-0.478079	1.218835
C18	0.268586	0.859422	1.216285
C19	0.471535	1.508431	0.000000
C20	0.268586	0.859422	-1.216285
C21	-0.149526	-0.478079	-1.218835
C22	-0.358736	-1.147682	0.000000
Cl7	-0.404753	-1.294156	2.727416
Cl8	0.546764	1.749005	2.678933
F9	0.869289	2.779679	0.000000
Cl10	-0.404753	-1.294156	-2.727416
Cl11	-0.876723	-2.805404	0.000000
Cl12	0.546764	1.749005	-2.678933

17

Table 3 entry 10 sigma-complex

C1	-0.765066	1.381368	-0.399929
C2	-2.088215	2.016847	-0.246915
C3	-3.126860	0.996070	-0.005401
C4	-2.857257	-0.335504	0.250595
C5	-1.545836	-0.851875	0.199410
C6	-0.514088	0.047195	-0.139254
Cl7	-4.774151	1.620670	0.018595
Cl8	-1.206896	-2.530952	0.589202
F10	-2.051142	2.950404	0.883971
O11	-2.405644	2.832765	-1.371063
C12	-2.629716	4.233134	-1.189198
H13	-2.860797	4.618172	-2.186889
H14	-3.476470	4.433010	-0.526102
H15	-1.742182	4.745552	-0.806682
Cl15	1.131054	-0.563667	-0.266773
Cl16	-4.183471	-1.432512	0.615099
Cl17	0.527671	2.486819	-0.861617

12

Table 3 entry 11 starting material

C17	3.260610	-1.597362	-0.093242
C18	2.165594	-2.353585	0.319373
C19	0.905272	-1.759673	0.314665
C20	0.727722	-0.436090	-0.092256
C21	1.838640	0.316315	-0.506007
C22	3.113735	-0.271590	-0.505219
F7	4.470729	-2.154266	-0.093154
Cl8	2.366622	-3.996464	0.827407
F9	-0.149206	-2.472601	0.707408
Cl10	1.637268	1.960713	-1.014539
Cl11	4.532511	0.594955	-1.002319
Cl12	-0.877687	0.221946	-0.065956

17

Table 3 entry 11 sigma-complex

C1	-0.761196	1.393452	-0.394307

C2	-2.096110	2.017920	-0.247185
C3	-3.115348	0.985834	-0.001215
C4	-2.821306	-0.334749	0.241483
C5	-1.518543	-0.848566	0.187138
C6	-0.493752	0.058364	-0.146146
Cl7	-4.794013	1.517301	0.061312
Cl8	-1.242369	-2.543215	0.547554
F10	-2.068886	2.958215	0.879849
O11	-2.433310	2.827334	-1.371990
C12	-2.592513	4.238840	-1.207613
H13	-2.833126	4.617563	-2.205483
H14	-3.412279	4.484656	-0.526305
H15	-1.675301	4.721020	-0.856806
Cl15	1.152088	-0.546700	-0.275178
F16	-3.829232	-1.188237	0.524331
Cl17	0.524850	2.512921	-0.848786

12

Table 3 entry 12 starting material

C17	3.268970	-1.612686	-0.090053
C18	2.169087	-2.366690	0.322816
C19	0.904628	-1.776577	0.320158
C20	0.740795	-0.456725	-0.087608
C21	1.845566	0.283069	-0.496651
C22	3.119047	-0.287209	-0.501434
F7	4.481355	-2.166672	-0.090562
Cl8	2.367027	-4.012828	0.832787
F9	-0.165798	-2.470041	0.709165
F10	1.669334	1.546765	-0.883960
Cl11	4.492921	0.640893	-1.010963
F12	-0.475686	0.099552	-0.086205

17

Table 3 entry 12 sigma-complex

C1	-0.900996	1.512974	-0.356062
C2	-2.267787	2.022134	-0.185791
C3	-3.192065	0.901912	0.019199
C4	-2.784074	-0.403160	0.231962
C5	-1.448935	-0.799555	0.149516
C6	-0.521845	0.214104	-0.160433
Cl7	-4.916047	1.276623	0.086192
Cl8	-0.936558	-2.434640	0.519253
F10	-2.298224	2.937929	0.997577
O11	-2.706464	2.857853	-1.246495
C12	-2.321109	4.235716	-1.229806
H13	-2.656096	4.646523	-2.186593
H14	-2.805944	4.779243	-0.414691
H15	-1.237950	4.359280	-1.149561
F15	0.784214	-0.111920	-0.305753
F16	-3.714892	-1.341580	0.516657
F17	0.050616	2.421574	-0.743175

12

Table 3 entry 13 starting material

C17	3.266126	-1.611021	-0.088996

C18	2.167084	-2.365242	0.323187
C19	0.903406	-1.773933	0.319522
C20	0.728348	-0.450877	-0.087287
C21	1.842779	0.282533	-0.495619
C22	3.116692	-0.286404	-0.500860
F7	4.476775	-2.164210	-0.089755
Cl8	2.368341	-4.011567	0.831642
F9	-0.152429	-2.483637	0.710893
F10	1.688261	1.545473	-0.886356
Cl11	4.492539	0.638468	-1.011449
Cl12	-0.849138	0.270540	-0.085375

17
Table 3 entry 13 sigma-complex

C1	-0.748644	1.378994	-0.410019
C2	-2.061072	2.035056	-0.261515
C3	-3.103733	1.022307	-0.003791
C4	-2.840649	-0.306712	0.238047
C5	-1.549028	-0.845927	0.176635
C6	-0.521595	0.044565	-0.159908
Cl7	-4.772406	1.587031	0.058410
Cl8	-1.236771	-2.538597	0.504382
F10	-2.011476	2.984831	0.858438
O11	-2.363815	2.850581	-1.394286
C12	-2.671772	4.233788	-1.208436
H13	-2.898233	4.614093	-2.209009
H14	-3.545121	4.383935	-0.566566
H15	-1.825640	4.793216	-0.798592
F15	0.733673	-0.442192	-0.262907
F16	-3.864154	-1.141302	0.522031
Cl17	0.611760	2.410948	-0.851271

In vacuo Energies for entries 1–12, Table 1

All energies are given in Hartrees

Table 1 entry 1 starting material −1104.786684
Table 1 entry 1 sigma-complex −1161.285384

Table 1 entry 2 starting material −1005.568989
Table 1 entry 2 sigma-complex −1062.071964

Table 1 entry 3 starting material −661.245519
Table 1 entry 3 sigma-complex −717.744772

Table 1 entry 4 starting material −744.412362
Table 1 entry 4 sigma-complex −800.921545

Table 1 entry 5 starting material −1104.788167
Table 1 entry 5 sigma-complex −1161.292582

Table 1 entry 6 starting material −661.175833

Table 1 entry 6 sigma-complex −717.700012

Table 1 entry 7 starting material −982.215014
Table 1 entry 7 sigma-complex −1038.733528

Table 1 entry 8 sigma-complex −1038.736332 (the energy for the starting material for this entry is the same as for entry 7)

Table 1 entry 9 starting material −1220.036013
Table 1 entry 9 sigma-complex −1276.546771

Table 1 entry 10 starting material −661.252215
Table 1 entry 10 sigma-complex −717.759747

Table 1 entry 11 sigma-complex −1276.569941 (the energy for the starting material for this entry is the same as for entry 9)

Table 1 entry 12 sigma-complex −1161.283261

In vacuo Energies for entries 1-8, Table 2

All energies are given in Hartrees

Table 2 entry 1 starting material −866.913343
Table 2 entry 1 sigma-complex −923.412125

Table 2 entry 2 starting material −1303.199890
Table 2 entry 2 sigma-complex −1359.666449

Table 2 entry 3 starting material −966.137849
Table 2 entry 3 sigma-complex −1022.647704

Table 2 entry 4 starting material −813.604666
Table 2 entry 4 sigma-complex -870.112817

Table 2 entry 5 starting material −1303.145967
Table 2 entry 5 sigma-complex −1359.666774

Table 2 entry 6 starting material −820.603745
Table 2 entry 6 sigma-complex −877.107671

Table 2 entry 7 starting material −813.610950
Table 2 entry 7 sigma-complex −870.116320

Table 2 entry 8 is the same as Table 1 entry 4, only the temperature and thus the experimental rate constants are different.

In vacuo Energies for entries 1-13, Table 3

All energies are given in Hartrees

Table 3 entry 1 starting material −629.158798
Table 3 entry 1 sigma-complex −744.284959

Table 3 entry 2 starting material −629.153522
Table 3 entry 2 sigma-complex −744.286826

Table 3 entry 3 starting material −629.159471
Table 3 entry 3 sigma complex −744.293485

Table 3 entry 4 starting material −728.374142
Table 3 entry 4 sigma complex −843.512456

Table 3 entry 5 starting material −1187.954958
Table 3 entry 5 sigma-complex −1303.102151

Table 3 entry 6 starting material −827.587708
Table 3 entry 6 sigma-complex −942.732321

Table 3 entry 7 sigma-complex -843.516875 (the energy for the starting material for this entry is the same as for entry 4)

Table 3 entry 8 sigma-complex -1303.105423 (the energy for the starting material for this entry is the same as for entry 5)

Table 3 entry 9 sigma-complex -1303.108509 (the energy for the starting material for this entry is the same as for entry 5)

Table 3 entry 10 starting material −2629.402554
Table 3 entry 10 sigma-complex −2744.566854

Table 3 entry 11 starting material −2269.046333
Table 3 entry 11 sigma-complex −2384.209046

Table 3 entry 12 starting material −1548.321946
Table 3 entry 12 sigma-complex −1663.480767

Table 3 entry 13 starting material −1908.688704
Table 3 entry 13 sigma-complex −2023.850269

Sigma Stability values for reaction series A calculated with a larger basis set

Reaction series A. Sigma Stability (SS) values for the same entries as in Table 1. in the main text, but calculated with a larger basis set including diffuse functions (6-31+G(d,p) with effective core potential on heavy atoms).

Entry	Reactant (Fig 1.)	Position of amination	SS (kcal mol^{-1}) water
1	**1**	2	10.73
2	**2**	2	10.38
3	**3**	Equal	7.27
4	**4**	4	5.38
5	**5**	4	6.35
6	**6**	4	1.04
7	**7**	2	3.04
8	**7**	4	2.19
9	**8**	2	0.09
10	**9**	4	−1.71
11	**8**	4	−0.28

Zero point energies for entries 1–11, Table 1

Entry	Reactant (Fig 1.)	Zero point energy for the sigma-complex (kcal/mol)	Zero point energy for the aromatic starting material (kcal/mol)	Difference between zero point energies for the sigma-complex and the corresponding starting material (kcal/mol)
1	**1**	53.942	29.620	24.322
2	**2**	59.042	34.685	24.357
3	**3**	52.541	28.175	24.366
4	**4**	55.088	30.511	24.577
5	**5**	54.379	29.655	24.724
6	**6**	52.305	27.771	24.534
7	**7**	63.111	38.524	24.587
8	**7**	63.430	38.524	24.906
9	**8**	71.429	46.566	24.863
10	**9**	53.319	28.503	24.816
11	**8**	71.756	46.566	25.190
